# The Gene-Drug Duality: Exploring the Pharmacogenomics of Indigenous Populations

**DOI:** 10.3389/fgene.2021.687116

**Published:** 2021-09-20

**Authors:** Shivashankar H. Nagaraj, Maree Toombs

**Affiliations:** ^1^Centre for Genomics and Personalised Health, School of Biomedical Sciences, Faculty of Health, Queensland University of Technology, Brisbane, QLD, Australia; ^2^School of Public Health, Faculty of Medicine, The University of Queensland, Herston, QLD, Australia

**Keywords:** pharmacogenomics, indigenous health, personalized medicine, chronic disease, adverse drug reaction

## Abstract

While pharmacogenomic studies have facilitated the rapid expansion of personalized medicine, the benefits of these findings have not been evenly distributed. Genomic datasets pertaining to Indigenous populations are sorely lacking, leaving members of these communities at a higher risk of adverse drug reactions (ADRs), and associated negative outcomes. Australia has one of the largest Indigenous populations in the world. Pharmacogenomic studies of these diverse Indigenous Australian populations have been hampered by a paucity of data. In this article, we discuss the history of pharmacogenomics and highlight the inequalities that must be addressed to ensure equal access to pharmacogenomic-based healthcare. We also review efforts to conduct the pharmacogenomic profiling of chronic diseases among Australian Indigenous populations and survey the impact of the lack of drug safety-related information on potential ADRs among individuals in these communities.

## Introduction

Pharmacogenomics, the “science of personalized medicine,” is a branch of genetics research that focuses on predicting a given individual’s responses to specific therapeutic drugs. The term “pharmacogenetics” was coined in 1959 by the German geneticist Friedrich Vogel to describe studies of individual genes and their impacts on drug activity (Kalow, 2006). Over time, pharmacogenetics evolved into the field of pharmacogenomics owing to the realization that most drug responses are multifactorial (Kalow, 2006).

The recent development of more potent and efficacious medications has highlighted the extreme variability in drug responses among individuals, leading to potentially life-threatening adverse drug reactions (ADRs), underscoring the need for a personalized approach to medicine. Pharmacogenomics studies seek to elucidate the genetic factors that influence a given individual’s responses to drugs and susceptibility to ADRs. By better understanding these factors, clinicians can more safely deliver effective medications tailored to a given individual’s genetic makeup (Kalow, 2006; Crews et al., 2012).

While there have been remarkable advancements in global genomic research during the last three decades, Indigenous populations have been severely underrepresented in most national and international pharmacogenomic studies (Hudson et al., 2020). Consequently, extant genomic data may not be relevant to these Indigenous individuals. Issues of trust, accountability, transparency, equity, lack of ethical frameworks, community outreach, and proper research guidelines have historically undermined global Indigenous genetic research efforts (Anderson et al., 2016; Hudson et al., 2020).

Australia has one of the largest Indigenous populations in the world, with Indigenous individuals from culturally and ancestrally distinct communities and over 250 different language groups comprising approximately three percent of the total Australian population (Australian Bureau of Statistics, 2021). Pharmacogenomic studies of these diverse Indigenous Australian populations have been hampered by several issues, including ethical concerns and lack of transparency resulting in the introduction of moratoriums on Indigenous genetic research by academic institutions such as the Australian National University in the 1990s (Claw et al., 2018; Australian Human Rights Commission, 2021). To improve knowledge regarding the pharmacogenomic landscape of Indigenous Australian populations, an Australian Indigenous reference genome is being created. Such a reference will facilitate the detection of rare unmapped variants unique to this population that cannot be identified using standard non-Indigenous population-based reference genomes. Herein, we review efforts to conduct the pharmacogenomic profiling of chronic diseases among Australian Indigenous populations and survey the potential impact of a lack of drug safety-related information on ADR risk among these communities.

## Pharmacogenomic Variants and Drug Responses

Drug response variability may be attributable to genetic differences between individuals resulting in variations in drug pharmacokinetics and pharmacodynamics (Lauschke et al., 2019). Germline pharmacogenomic biomarkers are primarily associated with allelic variants linked to variations in drug distribution, absorption, metabolism, and elimination (Lauschke et al., 2019; Ahsan et al., 2020). Differences in drug responses can also arise as a consequence of human leukocyte antigen (HLA) and drug target gene variants (Ahsan et al., 2020). Information regarding these variants can guide efforts to modulate drug efficacy and control ADR incidence (Lauschke et al., 2019; Ahsan et al., 2020). To effectively catalog known allelic variants associated with specific drug responses, the widely utilized “star” (^∗^) allele nomenclature system was developed. Under this system, gene names are appended with a number that denotes a particular allele or haplotype, with ^∗^ being used to designate the variant allele (e.g., CYP2C19^∗^2). This facilitates consistency among pharmacogenomic studies and clinical groups, ensuring the reliable classification of therapeutically relevant variants (Gaedigk et al., 2018). Currently, the ability to predict therapeutic outcomes and ADR incidence is of key importance in the oncology therapeutic space (Wheeler et al., 2013). To date, regulatory approval for the treatment of multiple cancers has been given to six small molecule inhibitors of epigenetic modifiers (epidrugs) with more under investigation (Yao et al., 2012; Wheeler et al., 2013). Significant variations are also observed in individual responses to psychiatric medications (Butler, 2018). A pharmacogenomic approach to the treatment of mental disorders would thus represent an efficacious means of reducing ADR incidence.

While early pharmacogenomic studies were reliant upon the targeted assessment of specific genotypic variations, recent advances in sequencing and bioinformatics have enabled comprehensive surveys of the pharmacogenomic landscape in specific populations. The Clinical Pharmacogenetics Implementation Consortium (CPIC) has published stringent guidelines for pharmacogenomics studies and for the high-quality annotation of datasets aimed at standardizing results from genome-wide association studies (GWAS; Manichaikul et al., 2010; Caudle et al., 2014). The CPIC currently lists 94 drug-dosage guidelines based upon pharmacogenomic findings, and the US Food and Drug Administration (FDA), 2021 has included pharmacogenomic label information on 372 drugs. As genome sequencing costs continue to decline and the value of personalized medicine becomes more fully realized, these tools will serve as the foundation for expanded pharmacogenomic medicine and guided prescription efforts (Supplementary Table 1).

## Population Pharmacogenomics

Individual drug responses vary in relation to gene polymorphisms with different ethnic distributions (Ramamoorthy et al., 2015). Differing ethnicities may impact genetic polymorphisms, leading to population-specific differences in drug responses. For example, African Americans are poor responders to angiotensin-converting enzyme inhibitors and β-blockers (Palleria et al., 2013), while patients with Asian ancestry are prescribed lower statin drug doses due to a higher susceptibility to statin-associated myopathy (Brewster and Seedat, 2013). The impact of co-medication depends on drug-drug interactions, concentrations of inhibitors or inducers, and drug half-lives. Potential factors regulating this process may be intrinsic (genetics, metabolism, and elimination) or extrinsic (diet, environmental exposure, and sociocultural differences) (Ramamoorthy et al., 2015). A high frequency (∼4,000-fold higher than Caucasian populations) of homozygous silent mutations in the butyrylcholinesterase gene (BChE; responsible for apnea, respiratory failure, and prolonged paralysis after succinylcholine or mivacurium treatment) has been reported in the Arya Vysya community in India (Liao, 2007). Earlier studies of Eskimos and Persian Jews also found a high prevalence of the autosomal recessive BChE allele mutation (Scott and Wright, 1976; David et al., 2015), resulting in increased incidence of pseudocholinesterase deficiency and likely conferring cardiovascular protection to these high-fat-consuming populations (Kaback et al., 2010). The HLA-B^∗^1502 allele, frequently found among members of the Han Chinese population, confers an elevated risk of Stevens-Johnson syndrome and associated severe toxic epidermal necrolysis when treated with carbamazepine (Pandit et al., 2011). However, despite such distinct pharmacogenomic variations, drug development programs rarely include individuals from these communities thus contributing to the paucity of data on Indigenous populations around the world in the context of genetic research.

## Pharmacogenomics of Australian Indigenous Populations

Australian Indigenous populations face a far greater burden of chronic diseases, substantially decreased life expectancy, and poorer overall general health relative to the non-Indigenous population, consistent with findings for Indigenous populations in other nations (Table 1; Thynne and Gabb, 2016; Beks et al., 2019). Most Indigenous Australians suffer from ≥1 chronic diseases (Tucci, 2011; Thynne and Gabb, 2016; Beks et al., 2019). Chronic diseases with a strong environmental and behavioral etiology, such as cardiovascular disease, hypertension, diabetes, obesity, chronic kidney disease, and depression, contribute to 80% of the mortality gap between Indigenous and non-Indigenous Australians under the age of 75 years (Tucci, 2011; Anderson et al., 2016). Kidneys from the Australian Indigenous individuals with no known kidney disease have been shown to have 30% fewer and significantly larger glomeruli than those from non-Indigenous individuals (Hoy et al., 2017; Thomson et al., 2019). Differences in the lipid profiles of Indigenous Australians relative to those of other Australians have also been observed, potentially influencing the efficacy of certain therapeutics (Hoy et al., 2017; Beks et al., 2019). Genetic predispositions have been implicated as factors influencing chronic mental health issues suffered by Indigenous Australians (Hoy et al., 2017; Das et al., 2018; Thomson et al., 2019). At least 20% of the 121 FDA-recognized pharmacogenetic markers are considered to be relevant in clinical practice involving psychiatric drugs (Currid and Mutsatsa, 2013; Butler, 2018; Nasir et al., 2018). Gene analytics and profiling programs have also shown associations between abnormal gene signaling networks and psychiatric illnesses like schizophrenia, bipolar disorder, and autism spectrum disorders (Butler, 2018). In Australia, little progress has been made in advancing the current understanding of these chronic diseases and mental disorders in Indigenous populations or improving the outcomes of affected individuals. More genetic epidemiological research must be conducted to properly address health outcomes among these Indigenous communities. A potential genetic predisposition to specific ADRs due to differences in allele frequencies of cytochrome P450 variants has been described in some earlier studies (Rheault et al., 2019). For example, the CYP2C19 and CYP2D6 genes in remote north western Australian Indigenous populations differ significantly from those observed in Australians of European ancestry, whereas these frequencies were similar to those observed in East Asian populations (Pandit et al., 2011; Tucci, 2011; Currid and Mutsatsa, 2013; Thynne and Gabb, 2016; Hoy et al., 2017; Das et al., 2018; Nasir et al., 2018; Beks et al., 2019; Rheault et al., 2019; Thomson et al., 2019). However, there remains a paucity of data linking ADRs to genetic predispositions in clinical settings, underscoring the urgent necessity of including the assessment and management of potential ADRs in any comprehensive healthcare program (Rheault et al., 2019).

**TABLE 1:** Chronic diseases in Australian indigenous populations.

Illness	Drugs with PGx information	PGx genes involved	Available guidelines	References
Chronic Kidney disease, Hypertension	ACE inhibitors, Losartan, Allopurinol	ACE, ABCB1, CYP2C9, CYP2D6, CYP3A4, CYP3A5, HLA-B	Allopurinol-HLA-B-CPIC	Saito et al., 2016
Coronary heart disease	Vitamin K antagonists, Platelet aggregation inhibitors, Selective Beta blockers, Antiarrhythmics, HMG CoA reductase inhibitors	ACE, ABCB1, CYP2C9, CYP2C19, CYP3A4, CYP3A5, CYP4F2, SLCO1B1, VKORC1	Vitamin K antagonists-CYP2C9, CYP4F2, VKORC1-CPIC, Platelet aggregation inhibitors-CYP2C19-CPIC, HMG CoA reductase inhibitors-SLCO1B1-CPIC	Scott et al., 2013; Ramsey et al., 2014; Johnson et al., 2017
Diabetes	Sulphonamides	CYP2C9	NA	–
Pain	NSAIDS, halogenated Anesthetics, Opioids	CACNA1S, CYP2C8, CYP2C9, CYP2D6, RYR1	NSAIDS-CYP2C9-CPIC, Anesthetics- CACNA1S, RYR1-CPIC, Codeine-CYP2D6-CPIC	Crews et al., 2014; Gonsalves et al., 2019; Theken et al., 2020
Mental disorders	Selective serotonin reuptake inhibitors (SSRI), Tricyclic anti-depressants (TCA), Haloperidol, Diazepines, Risperidone, Atomoxetine	CYP2C19, CYP2D6, CYP3A4	SSRI, TCA-CYP2C19, CYP2D6-CPIC,Atomoxetine-CYP2D6-CPIC	Hicks et al., 2015; Hicks et al., 2017; Brown et al., 2019
Cancer	Purine and Pyrimidine analog, platinum compounds, PEG-interferon-alpha	DPYD, IFNL3, NUDT15, TPMT, XPC	Purines-NUDT15, TPMT-CPIC, Pyrimidines-DPYD-CPIC, PEG-Iα-IFNL3-CPIC	Muir et al., 2014; Amstutz et al., 2018; Relling et al., 2019

## Indigenous Pharmacogenomic Research and the Ethical Dissemination of Information

Multiple genomic consortiums have attempted to enlist Indigenous participants to map genomic diversity across populations (Supplementary Table 2). However, most of these studies, including the Human Genome Diversity Project and the National Genographic Project, failed to adequately recruit and include Indigenous peoples due to a lack of a well-planned and inclusive ethical framework (Claw et al., 2018). The ongoing All of Us project—a research -project for gathering genetic, environmental, and lifestyle data from over one million US residents sponsored by the US National Institutes of Health, 2021—is currently attempting to engage in appropriate tribal consultations. However, despite initiatives to make genomic data more accessible, public trust across the diverse Indigenous communities is as yet lacking. As Bentley et al. (2017) stated in the context of genomic research in Africa, “public trust, oversight, and long-lasting relationships with communities who participate in genomic research are required to advance both data sharing, and diversity and inclusion—two major components of genomic research that must advance symbiotically for genomic research to benefit all.”

Genomic data are more sensitive than other types of health data for Indigenous communities as they can influence traditional and cultural beliefs and also affect identity claims for rights to land and other resources. Targeted policies to promote responsible conduct of research have been developed by some Indigenous communities. Research guidelines (Supplementary Table 3) such as the Human Heredity and Health in Africa (H3Africa) guidelines for community engagement in Africa and the Te Mata Ira guidelines for Genomic Research with Maori in New Zealand, help Indigenous communities to develop an optimistic outlook regarding the outcomes of genomic research. The Navajo Nation in the United States has begun to develop a culturally informed genetic research policy replacing the 16-year moratorium on genetic research. Two similar independent initiatives–the “Silent Genomes” in Canada and the “Aotearoa Variome” in New Zealand have undertaken research to collect and curate genomic variation databases to better understand and undertake translational genetic research (Caron et al., 2020). These systematic efforts to engage Indigenous communities in healthcare and genomic research represent the first steps toward bridging the genomic knowledge gap regarding the Indigenous populations of the world, with years if not decades worth of research yet to be completed. Recognizing and understanding the inherent right of Indigenous people to develop socio-economically and culturally is the starting block for relationship building and must be reflected in the research frameworks (Claw et al., 2018; Garrison et al., 2019). One approach toward faciliating a paradigm shift toward equitable benefit sharing would be to ensure that Indigenous people have control over the data from Indigenous populations, including digital sequence information. Notably, Indigenous scholars and policy makers are leading initiatives to improve access to genomic research in health care throughout Canada, New Zealand, Australia, and the United States (Claw et al., 2018; Garrison et al., 2019; Caron et al., 2020). These countries have endorsed the United Nations Declaration on the Rights of Indigenous Peoples (Garrison et al., 2019). Genomics has the potential to revolutionize the way health care is delivered in Australia. Current ongoing research programs at the state and national level are attempting to introduce genomics into the Australian healthcare system. It has been identified that Australian Indigenous people are at risk of being “left out” of these developments due to the inability of researchers to engage with Indigenous communities and conduct genomics research in such settings. Recently, the Indigenous Genomics Health Literacy Project (IG-HeLP), 2021 has been implemented to educate and inform the Australian Aboriginal and Torres Strait Islander consumers and health workers on the topics of DNA, genes, genetic health, genetic testing, and precision medicine. Such efforts will facilitate cultural inclusivity in genomic research and equitable access to the healthcare benefits of clinical genomics across Australia, and thereby, has the potential to shape Australia’s health future (GenetiQs Project, 2021; QIMR Berghofer, 2021).

## Conclusion

Multiple changes in the scientific community are required to ensure that Indigenous populations benefit from genomic research. One method is the development of an Indigenous reference genome for cataloging genetic variants. There are over 500 different clan groups or “nations” in Australia, each with distinctive cultures, beliefs and languages. Ideally, a minimum of one reference genome per clan is required for effective high quality variant calling and to understand their underlying genomic architecture (Shumate et al., 2020). Efforts are ongoing to increase the number of Indigenous researchers working in genomic sciences and to develop novel culturally safe guidelines. Initiatives such as the AHRA, 2021 National Indigenous Research(er) Capacity Building Network (IRNet) are creating opportunities to improve Indigenous pharmacogenomic health literacy, increasing the number of Indigenous researchers working in genomic science, developing novel culturally safe guidelines, policies, and procedures for bridging the genomic divide, and respecting the rights, interests, and opinions of Indigenous participants in relation to their genomic data (Claw et al., 2018; Shumate et al., 2020; NHMRC, 2021). A proactive and comprehensive approach to address drug safety in Indigenous populations in the post-marketing space is also urgently required.

Pharmacogenomic variations remain understudied in Indigenous Australian populations and may have important implications for drug efficacy and ADR risk. This apparent failure, despite numerous funded Indigenous health programs functioning at a national level and under the “Closing the Gap” policy, has been attributed to issues of methodology relating to culturally appropriate acceptable research approaches accounting for the diversity of Indigenous populations, short government funding cycles, and no community ownership and consultation (Anderson et al., 2016; Rheault et al., 2019; Hudson et al., 2020). Initiatives like the National Center for Indigenous Genomics have been established to facilitate participatory, consensual, and ethical genomic research which may help to gradually close this chronic disease gap affecting Indigenous Australian communities, thereby advancing pharmacogenomic research.

## Author Contributions

SN created Tables and Figures. Both authors conceived and prepared, read and approved the final manuscript.

## Conflict of Interest

The authors declare that the research was conducted in the absence of any commercial or financial relationships that could be construed as a potential conflict of interest.

## Publisher’s Note

All claims expressed in this article are solely those of the authors and do not necessarily represent those of their affiliated organizations, or those of the publisher, the editors and the reviewers. Any product that may be evaluated in this article, or claim that may be made by its manufacturer, is not guaranteed or endorsed by the publisher.
