# Supplementary material for: The Gene-Drug Duality: Exploring the Pharmacogenomics of Indigenous Populations
Source: Front Genet. 2021 Sep 20;12:687116. doi: 10.3389/fgene.2021.687116 (PMC8488351; doi:10.3389/fgene.2021.687116)
Supplement: Supplementary file 1 [file Table_1.DOCX]

**The Gene-Drug Duality: Exploring the Pharmacogenomics of Indigenous Populations**

Shivashankar H Nagaraj and Maree Toombs

**Supplementary Table 1:** Selected genomic databases

| **Database** | **URL** |
| --- | --- |
| NCBI Genome | https://www.ncbi.nlm.nih.gov/genome |
| Ensembl Genome | http://ensemblgenomes.org/ |
| GWASCentral | https://www.gwascentral.org/ |
| DGV Databaase of genomic variation | http://dgv.tcag.ca/dgv/app/home |
| ENCODE Encyclopedia of DNA elements | https://www.encodeproject.org/ |
| 1000 Genomes Project | http://www.internationalgenome.org/ |
| Simons genome diversity | https://www.simonsfoundation.org/simons-genome-diversity-project/ |
| TCGA The Cancer Genome Atlas | https://portal.gdc.cancer.gov/ |
| DDBJ DNA Database of Japan (DDBJ) | https://www.ddbj.nig.ac.jp/ |
| EMBL Nucleotide Sequence Database | https://www.ebi.ac.uk/ena/browser/home |
| GenBank Database | https://www.ncbi.nlm.nih.gov/genbank/ |
| dbSNP (Database of single nucleotide polymorphism) | https://www.ncbi.nlm.nih.gov/snp/ |
