# Supplementary material for: The Gene-Drug Duality: Exploring the Pharmacogenomics of Indigenous Populations
Source: Front Genet. 2021 Sep 20;12:687116. doi: 10.3389/fgene.2021.687116 (PMC8488351; doi:10.3389/fgene.2021.687116)
Supplement: Supplementary file 2 [file Table_2.DOCX]

**The Gene-Drug Duality: Exploring the Pharmacogenomics of Indigenous Populations**

Shivashankar H Nagaraj and Maree Toombs

**Supplementary** **Table 2** Genomic consortiums recruiting Indigenous populations

| **Name of Study** | **Country** | **Completed/Ongoing** | **Key findings** |
| --- | --- | --- | --- |
| Translating genotype data of 44,000 biobank participants into clinical pharmacogenetic recommendations: challenges and solutions [1] | Estonia | Completed | Microarrays can serve as a feasible solution for PGx testing. Estimates 50 PGx advice for 1000 prescribed doses. |
| Pharmacogenetics at scale: An analysis of the UK Biobank [2] | UK | Ongoing | Highlights importance of understanding rare and deleterious variation in PGX implementation. 99.5% of individuals surveyed had atypical response to at least one drug with an average atypical response to 12 drugs. |
| Pharmacogenomic variation in DiscovEHR cohort[3, 4] | USA | Completed | Rare and novel clinically relevant PGx variants |
| EMERGE Network[5] | USA | Ongoing | PGx integration with Electronic Health records |
| China Kadoorie Biobank[6] | China | Ongoing | - |
| PGRN Japan, Japan Biobank[7, 8] | Japan | Ongoing | HLA and CYP2C9 risk alleles for phenytoin adverse events in Japanese individuals |
| Ubiquitous-PGx, PREPARE[9] | European Union | Ongoing | - |
| Pharmacogenomic variation in 1000 genomes [10, 11] | - | Completed | Population specific differences between and within different populations highlighted |
| Pharmacogenomic variation in gnomAD genomes [3, 12] | - | Completed | Population specific differences between and within different populations highlighted |
| Pharmacogenomic survey of Qatari populations[13] | Qatar | Completed | PGx variation landscape in Qatari populations, Clinical implications |
| Genetic epidemiology of pharmacogenetic variants in South East Asian Malays[14] | Malaysia | Completed | PGx variation landscape in SEA Malays, Clinical implications |
| Population Pharmacogenomics for Precision Public Health in Colombia[15] | Colombia | Completed | PGx variation landscape in two Colombian populations, Public health implications |
