# Supplementary material for: The Gene-Drug Duality: Exploring the Pharmacogenomics of Indigenous Populations
Source: Front Genet. 2021 Sep 20;12:687116. doi: 10.3389/fgene.2021.687116 (PMC8488351; doi:10.3389/fgene.2021.687116)
Supplement: Supplementary file 3 [file Table_3.DOCX]

**The Gene-Drug Duality: Exploring the Pharmacogenomics of Indigenous Populations**

Shivashankar H Nagaraj and Maree Toombs

**Supplementary Table 3**: Global Indigenous research guidelines/policies

| Guideline/policy name | Institution | Date of creation | Country/target population |
| --- | --- | --- | --- |
| NCIG Governance framework [16] | National Centre for Indigenous Genomics | 23/07/18 | Australia |
| Ethical conduct in research with Aboriginal and Torres Strait Islander Peoples and communities: Guidelines for researchers and stakeholders [17] | National Health and Medical Research Council, Australia | August 2018 | Australia |
| Te Mata Ira: Guidelines for Genomic Research with Māori [18] | Te Mata Hautū Taketake – Māori & Indigenous Governance Centre, New Zealand | October 2016 | New Zealand |
| Guidelines for Researchers on Health Research Involving Māori [19] | Health Research Council, New Zealand | 2010 | New Zealand |
| Chapter 9: RESEARCH INVOLVING THE FIRST NATIONS, INUIT AND MÉTIS PEOPLES OF CANADA, Tri-Council Policy Statement 2: Ethical Conduct for Research Involving Humans  [20] | Government of Canada | 2018 | Canada |
| Subpart A—Basic HHS Policy for Protection of Human Research Subjects [21] | US Department of Healtand Human Service | 19/06/2018 | United States of America |
